# Core intended learning outcomes for tackling health inequalities in undergraduate medicine

**DOI:** 10.1186/s12909-015-0342-1

**Published:** 2015-04-02

**Authors:** Andrea E Williamson, Richard Ayres, Jessica Allen, Una Macleod

**Affiliations:** 1 General Practice and Primary Care, School of Medicine, MVLS, University of Glasgow, 1 Horselethill Road, Glasgow, G12 9LX UK; 2 Plymouth Peninsula Schools of Medicine and Dentistry, University of Plymouth, Plymouth, UK; 3 UCL Institute of Health Equity, University College London, London, UK; 4 Centre for Health and Population Sciences, Hull York Medical School, York, UK

**Keywords:** Undergraduate medical curriculum, Intended learning outcomes, Tackling health inequalities, Expert consensus

## Abstract

**Background:**

Despite there being a concerted effort in recent years to influence what doctors can do to tackle health inequalities in the UK, there has been limited policy focus on what undergraduate students need to learn at medical school in preparation for this. This project led by members of the Health Inequalities Group of the Royal College of General Practitioners in collaboration with the Institute of Health Equity, University College London sought to fill this gap.

**Discussion:**

We conducted a Delphi poll using our teaching and stakeholder networks. We identified 5 areas for learning focusing on key knowledge and skills. These were population concepts, health systems, marginalised patient groups, cultural diversity and ethics.

**Summary:**

These intended learning outcomes about health inequalities represent the best available evidence to date for colleagues seeking to develop core undergraduate medical curricula on the topic.

**Electronic supplementary material:**

The online version of this article (doi:10.1186/s12909-015-0342-1) contains supplementary material, which is available to authorized users.

## Background

Inequalities in health are persistent and pervasive, and health services are often not targeted at those who need them most [1,2] Building on the World Health Organisation Commission on the social determinants of health [3] the Marmot review in England made the case for doctors to engage with the underlying social determinants of health [4] and led to a further report on what health professionals can do to tackle health inequalities [5]. The Royal College of General Practitioners, and the Royal College of Physicians have also published reports about the role of doctors in reducing inequalities [6,7], and the Academy of Medical Royal Colleges has published a consensus document on a health inequalities curriculum for postgraduate specialist training [8]. However there has been no specific policy attention paid to what medical students should be learning in UK medical schools to prepare them to take up these challenges when they graduate.

In Aug 2013, the Health Inequalities Standing Group (HISG), of the UK Royal College of General Practitioners (RCGP), in collaboration with the Institute for Health Equity (IHE), University College London sought to fill this gap. HISG is a co-opted committee of general practitioners and patient representatives who have clinical, teaching or research expertise in tackling health inequalities and the health care of marginalised patient groups in the UK. It has an RCGP remit to tackle health inequalities through influencing policy and practice and one of its strategic aims is a focus on undergraduate medical education. The IHE works collaboratively with a range of partners who are working to tackle health inequalities.

The aim of the project was to produce guidance based on experienced consensus and that medical educators working in the UK medical schools would find useful in helping develop teaching and learning about health inequalities in their unique medical school and geographical contexts.

A “Delphi” poll of medical educators across each of the 32 medical schools in the UK and 20 stakeholder organisations (Royal Colleges, British Medical Association, General Medical Council) was conducted. A starter list of intended learning outcomes (ILO’s) mapped to Tomorrows Doctors 2009 which describes the competency outcomes guiding UK medical school curricula by the UK General Medical Council was compiled by HISG members with representation from the student body Medsin (Additional file 1).

These two questions informed the project’s development:

What are the core competencies we wish new medical graduates to have so they are equipped for working as an FY1 (doctor in training in the UK) and for lifelong learning?

What are the knowledge skills and attributes that UK medical students interested in learning more about health inequalities could cover?

Using teaching network contacts, snow ball sampling and an online survey, these ILOs were developed using an iterative process of two Delphi rounds (Additional file 2) into a consensus statement for core learning, additional learning and current good practice examples from around the UK. Twenty one medical educators representing 19 of the 32 medical schools in the UK took part. This means the core curriculum that follows represents this particular perspective and does not include other stakeholders, for example patients who experience health inequalities themselves. Participants were encouraged to suggest ILOs that should be removed, modified or new ones that should be added at both stages of the process.

## Discussion

The core intended learning outcomes that were agreed from this process cover five areas focusing on key knowledge and skills that provide strong foundations for medical school graduates entering the workforce.

**Core intended learning outcomes for tackling health inequalities**

1. ***Health inequalities-population concepts***
  1.1. Define the concept of health inequalities using examples from the UK and globally
  1.2. Understand the concepts behind the social determinants of health
  1.3. Describe the evidence base for health inequalities aspects of common conditions such as obesity, diabetes, cardio-vascular disease and mental health in the UK and globally
  1.4. Be able to describe difference between area level indicators of socioeconomic status (SES) and individual level indicators of SES (e.g. obeso-genic environment and dietary intake)
  1.5. Be able to take a targeted social history from patients
2. ***Health inequalities- health systems impact***
  2.1. Describe how health policy, health care systems and the wider context of society impacts on health inequalities
  2.2. Examine the inverse care law using examples from the UK and globally
  2.3. Understand the key principles of primary health care and its role in reducing health inequalities
3. ***Marginalised patient groups***
  3.1. Be able to describe the major problems of health and health care delivery for marginalised patient groups in the UK (e.g. homeless persons, asylum seekers)
  3.2. Be able to communicate effectively with patients with special communication needs
  3.3. Describe the needs of economically deprived older people and young people in care
  3.4. Be able to take measures to safeguard children and other vulnerable persons
4. ***Cultural diversity***
  4.1. Define ‘cultural diversity’ and apply this definition with respect to clinical practice
  4.2. Critically appraise the use of key terms, such as race, ethnicity, culture, multiculturalism and inequalities of access to health care
  4.3. Be able to communicate effectively with patients from diverse backgrounds
  4.4. Evaluate institutional prejudices and how these relate to your own perspectives
5. ***Health inequalities-ethics***
  5.1. Be able to discuss and critique how the concept of a right to health impacts on health care delivery in the UK and elsewhere
  5.2. Understand Equality and Human Rights legislation and how it overlaps with health inequalities
  5.3. Be able to consider strategies for enacting the important advocacy role that doctors have
  5.4. Develop a generic approach to patients from diverse backgrounds, understanding that some patients require more input and advocacy than others
  5.5. Demonstrate empathy and compassion with all patients
  5.6. Respect the unique perspective of all patients
  5.7. Understand the impact your own beliefs and values may have on the care of patients

The first is population concepts since it is impossible to grasp the concept of health inequalities without understanding how health is distributed across society, how it is measured and what influences it. The second is health systems. The world concluded at Alma Ata in 1978 [9] that primary health care was the only way to achieve “health for all”. This is echoed in contemporary calls for universal health care [10]. How this is achieved or not by local and global health systems is a key curriculum area.

The third area relates to marginalised patient groups, for example homeless people [11], people with learning disabilities [12], and children [13]. What should professionals and services do to deliver effective care for these patient groups?

The fourth, cultural diversity, which include the competencies required to perform well as a doctor in modern globalised multi-cultural environments.

The fifth and most pervasive area is ethics. The Delphi respondents echoed strongly Zsuzsanna Jakab, WHO Regional Director for Europe who in her foreword to the WHO report on social determinants of health across Europe, said “health inequities offend against the human right to health and are unnecessary and unjust” [14]. Underpinning that is the principle of advocacy, that doctors can and should influence the circumstances in which their patients are “born, grow, live, work and age” [15]. This is now explicitly proposed by mainstream groups such as the British Medical Association. Doctors are encouraged to be advocates for change beyond their direct role as clinicians “locally, regionally, nationally and internationally” [16]. Equipping future doctors with the tools to do this is a major challenge for medical education.

Three of these areas (health systems, cultural diversity, and ethics) were included in the proposed core learning outcomes for Global Health published by the Global Health Learning Outcomes Working Party in 2012 [17], which is no surprise since tackling health inequalities is a central theme in global health. This consensus statement of core intended learning outcomes for tackling health inequalities builds on that work and now provides the best available evidence for medical educators focused on delivering learning about health inequalities in UK medical schools.

## Summary

We hope this consensus statement will serve two additional purposes. One is to inform medical educators working in other countries about this UK based health inequalities learning development. The second is to further strengthen the case for these topic areas and the wider global health learning agenda to be incorporated into undergraduate medical curricula. This will produce medical graduates equipped to recognise and tackle inequity in 21st century healthcare systems [18].

The full report which includes additional intended learning outcomes for students interested in learning more about health inequalities, and inspiring examples of existing good practice in the UK can be accessed at http://www.rcgp.org.uk/policy/rcgp-policy-areas/health-inequalities.aspx

## Ethical review

Not required. It’s a role of HISG to seek and contribute expert peer input through professional networks. Consent was sought and obtained by professionals taking part in the Delphi poll.

## Additional files

Additional file 1:
**DelphipollstarterlistwithGMCcompetencies.**
Additional file 2:
**Delphipollcollatedresults.**
