# Supplementary material for: Core intended learning outcomes for tackling health inequalities in undergraduate medicine
Source: BMC Med Educ. 2015 Apr 2;15:66. doi: 10.1186/s12909-015-0342-1 (PMC4389346; doi:10.1186/s12909-015-0342-1)
Supplement: Additional file 1: — DelphipollstarterlistwithGMCcompetencies. [file 12909_2015_342_MOESM1_ESM.pdf]

Delphi poll of UK undergraduate medical educators
Tackling health inequalities, learning for undergrad medical students in the UK

Name: **Role:** **Medical School:** **Email contact:** **Date:**

Thank you for taking the time to take part in this first round of this Delphi poll. There are 2 sections to review.
The first section asks about the competencies required by undergraduate medical students in UK medical schools. These are divided into those that you think should be core learning about health inequalities for ALL undergraduates in UK medical schools and those that you think could be considered additional learning for undergraduates who are interested in learning more (by undertaking student selected modules or electives).
The second section hopes you might be able to provide examples of good practice in teaching health inequalities already taking place in your medical school.

Section 1
Questions: What are the ***core competencies*** we wish all new medical graduates to have so they are equipped for working as an FY1 doctor and for life long learning?
What ***additional competencies*** might new medical graduates acquire if they have a particular interest in tackling health inequalities?

Please read through the statements below. They are ILOs mapped to the GMC's TD2009. Tick one of the boxes: **core** if you think all medical students should learn this, **additional** if you think its optional learning for interested students and **neither** if not relevant. Add comments if you wish about why you have made this choice or others about wording etc.

Domain	Intended learning outcome (ILO's)	Maps to Tomorrow's Doctor 2009 competencies [1]	core	additio nal	neither	Comments
Knowledge	Be able to define the concept of health	11(a) 11(f)				

[1] Purpose of mapping to Tomorrow's Doctors 2009 (TD 2009):
"The outcomes set out what the GMC expects medical schools to deliver" These GMC competencies are set out on page 7 and 8.

	inequalities using examples from the UK and globally					
	Outline the key analytical frameworks for understanding the social determinants of health	10 (a) (b) (c) (d) (e)<br>11 (a) (b) (d) (f) (g) (h) (j)				
	Discuss and critique how the concept of a right to health impacts on health care delivery in the UK and elsewhere	11 (d) (j)<br>23 ©				
	Be able to review one risk factor and one important disease topic from a social determinants of health and health inequalities perspective using population based	9 (c) (e) (g)<br>10 (c) (d)(e)<br>11 (b) (d) (f) (h) (i)<br>12 (a) (b) (c) (d)<br>22 (a) (b) (c)				

Name: **Role:** **Medical School:** **Email contact:** **Date:**

	analyses. (eg cardio-vascular disease) or harmful alcohol use)					
	Examine the inverse care law using examples from the UK and globally	11(d) (j)				
	Describe the major problems of health and health care delivery for marginalised patient groups in the UK (eg homeless persons, asylum seekers)	9 (c) (e) (g)<br>11 (b) (d) (f)<br>13 (a)<br>14 (a) (i)<br>22 (a) (b) (c)				
Skills	Be able to take a targeted social history from patients	13 (a)<br>15 (d) (g)				
	Communicate effectively with patients from diverse backgrounds	13 (a)<br>14 (a)<br>15 (b) (e) (f) (g)<br>20(d) (e) (f)<br>22 (a) (b) (c)				
	Communicate	14 (a)				

	effectively with patients with special communication needs	15 (b) (g)<br>20(d) (e) (f)<br>22 (a) (b) (c)				
	Be able to take measures to safeguard children and other vulnerable persons	14 (a) (i)<br>20 (e) (f)<br>22 (a) (b) (c)				
	Consider strategies for enacting the important advocacy role that doctors have	15 (h)<br>22 (d)				
Attributes(attitudes and values)	Respect the unique perspective of all patients	15 (b) (d) (e) (f) (g) (h)<br>20 (d) (e) (f)				
	Understand the impact your own beliefs and values may have on the care of patients	15 (b) (d) (e) (f) (g) (h)				

Delphi poll of UK undergraduate medical educators
Tackling health inequalities, learning for undergrad medical students in the UK

Name: **Role:** **Medical School:** **Email contact:** **Date:**

Question: Are there knowledge, skills or attributes that should be **core competencies** for medical graduates in the UK that we have **not yet described**?

Please use this space to tell us about them:

Question: Are there knowledge, skills and attributes that students **interested in additional learning** about tackling health inequalities that we have **not yet described**?

Please use this space to tell us about them:

Delphi poll of UK undergraduate medical educators
Tackling health inequalities, learning for undergrad medical students in the UK

Name: **Role:** **Medical School:** **Email contact:** **Date:**

Section 2

We would like to share **examples of existing good practice** in teaching about health inequalities with other medical educators. If you or colleagues in the medical school are already delivering learning about health inequalities and would like to tell us about it please summarise what you do here:

Name: **Role:** **Medical School:** **Email contact:** **Date:**

Tackling health inequalities maps to the following GMC TD2009 outcomes:

9 Apply psychological principles, method and knowledge to medical practice.
(c) Apply theoretical frameworks of psychology to explain the varied responses of individuals, groups and societies to disease.
(e) Discuss psychological aspects of behavioural change and treatment compliance.
(g) Identify appropriate strategies for managing patients with dependence issues and other demonstrations of self-harm.
10 Apply social science principles, method and knowledge to medical practice.
(a) Explain normal human behaviour at a societal level.
(b) Discuss sociological concepts of health, illness and disease.
(c) Apply theoretical frameworks of sociology to explain the varied responses of individuals, groups and societies to disease.
(d) Explain sociological factors that contribute to illness, the course of the disease and the success of treatment – including issues relating to health inequalities, the links between occupation and health and the effects of poverty and affluence.
(e) Discuss sociological aspects of behavioural change and treatment compliance.
11 Apply to medical practice the principles, method and knowledge of population health and the improvement of health and healthcare.
(a) Discuss basic principles of health improvement, including the wider determinants of health, health inequalities, health risks and disease surveillance.
(b) Assess how health behaviours and outcomes are affected by the diversity of the patient population.
(d) Discuss the principles underlying the development of health and health service policy, including issues relating to health economics and equity, and clinical guidelines.
(f) Evaluate and apply epidemiological data in managing healthcare for the individual and the community.
(g) Recognise the role of environmental and occupational hazards in ill-health and discuss ways to mitigate their effects.
(h) Discuss the role of nutrition in health.
(i) Discuss the principles and application of primary, secondary and tertiary prevention of disease.4
(j) Discuss from a global perspective the determinants of health and disease and variations in healthcare delivery and medical practice.
12 Apply scientific method and approaches to medical research.
(a) Critically appraise the results of relevant diagnostic, prognostic and treatment trials and other qualitative and quantitative studies as reported in the medical and scientific literature.
(b) Formulate simple relevant research questions in biomedical science, psychosocial science or population science, and design appropriate studies or experiments to address the questions.
(c) Apply findings from the literature to answer questions raised by specific clinical problems.
(d) Understand the ethical and governance issues involved in medical research.
13 The graduate will be able to carry out a consultation with a patient:
(a) Take and record a patient's medical history, including family and social history, talking to relatives or other carers where appropriate. treatment.
14 Diagnose and manage clinical presentations.

Name: **Role:** **Medical School:** **Email contact:** **Date:**

(a) Interpret findings from the history, physical examination and mental-state examination, appreciating the importance of clinical, psychological, spiritual, religious, social and cultural factors.

(i) Identify the signs that suggest children or other vulnerable people may be suffering from abuse or neglect and know what action to take to safeguard their welfare.

15 Communicate effectively with patients and colleagues in a medical context.

(b) Communicate clearly, sensitively and effectively with individuals and groups regardless of their age, social, cultural or ethnic backgrounds or their disabilities, including when English is not the patient's first language.

(d) Communicate appropriately in difficult circumstances, such as when breaking bad news, and when discussing sensitive issues, such as alcohol consumption, smoking or obesity.

(e) Communicate appropriately with difficult or violent patients.

(f) Communicate appropriately with people with mental illness.

(g) Communicate appropriately with vulnerable patients.

(h) Communicate effectively in various roles, for example, as patient advocate, teacher, manager or improvement leader.

20 The graduate will be able to behave according to ethical and legal principles. The graduate will be able to:

(d) Respect all patients, colleagues and others regardless of their age, colour, culture, disability, ethnic or national origin, gender, lifestyle, marital or parental status, race, religion or beliefs, sex, sexual orientation, or social or economic status. Graduates will respect patients' right to hold religious or other beliefs, and take these into account when relevant to treatment options.

(e) Recognise the rights and the equal value of all people
and how opportunities for some people may be restricted by others' perceptions.

(f) Understand and accept the legal, moral and ethical responsibilities involved in protecting and promoting the health of individual patients, their dependants and the public − including vulnerable groups such as children, older people, people with learning disabilities and people with mental illnesses.

22 Learn and work effectively within a multi-professional team.

(a) Understand and respect the roles and expertise of health and social care professionals in the context of working and learning as a multi-professional team.

(b) Understand the contribution that effective interdisciplinary teamworking makes to the delivery of safe and high-quality care.

(c) Work with colleagues in ways that best serve the interests of patients, passing on information and handing over care, demonstrating flexibility, adaptability and a problem-solving approach.

(d) Demonstrate ability to build team capacity and positive working relationships and undertake various team roles including leadership and the ability to accept leadership by others.

23 Protect patients and improve care.

(c) Understand the framework in which medicine is practised in the UK, including: the organisation, management and regulation of healthcare provision; the structures, functions and priorities of the NHS; and the roles of, and relationships between, the agencies and services involved in protecting and promoting individual and population health.

Delphi poll of UK undergraduate medical educators
Tackling health inequalities, learning for undergrad medical students in the UK

Name:	Role:	Medical School:	Email contact:	Date:
