# Supplementary material for: Core intended learning outcomes for tackling health inequalities in undergraduate medicine
Source: BMC Med Educ. 2015 Apr 2;15:66. doi: 10.1186/s12909-015-0342-1 (PMC4389346; doi:10.1186/s12909-015-0342-1)
Supplement: Additional file 2: — Delphipollcollatedresults. [file 12909_2015_342_MOESM2_ESM.pdf]

Delphi consensus project: devising a core curriculum for learning about health inequalities in UK undergraduate medicine

Here are the results from the 2 rounds of the Delphi poll.
TABLE 1 is the 'starter' ILO's we suggested and their subsequent votes.
TABLE 2 is the core ILO's suggested by Delphi participants and their subsequent vote.

The ILO's are highlighted by their themes to help you see where they move to
Health inequalities-population concepts
Health inequalities- health systems impact
Marginalised patient groups
Cultural diversity
Health inequaliites- ethics
The ILO's not highlighted have been moved into the additional ILOs category for collation.

TABLE 1

	Knowledge ILOs	Round 1 vote		Round 2 vote	Outcome
	Be able to define the concept of health inequalities using examples from the UK and globally	21	0	n/a	core
	Be able to discuss and critique how the concept of a right to health impacts on health care delivery in the UK and elsewhere	21	0	n/a	core
	Be able to review one risk factor and one important disease topic from a social determinants of health and health inequalities perspective using population based analyses (e.g. cardio-vascular disease or harmful alcohol use)	17	4	10/10	HISG discuss (duplicated?)
	Be able to describe the major problems of health and health care delivery for marginalised patient groups in the UK (e.g. homeless persons, asylum seekers)	19	2	9/10	core
	Be able to examine the inverse care law using examples from the UK and globally	16	4	8/10	core

Be able to outline the key analytical frameworks for understanding the social determinants of health	14	7	7/10	HISG discuss
**Skill ILO's**				
Be able to take a targeted social history from patients	20	0	n/a	core
Be able to communicate effectively with patients from diverse backgrounds	21	0	n/a	core
Be able to communicate effectively with patients with special communication needs	17	4	8/9	core
Be able to take measures to safeguard children and other vulnerable persons	19	2	9/9	core
Be able to consider strategies for enacting the important advocacy role that doctors have	17	3	7/9	core
**Attribute ILO's**				
Understand the impact your own beliefs and values may have on the care of patients	21	0	n/a	HISG discuss
Respect the unique perspective of all patients	20	1	9/9	core

Core Knowledge ILOs suggested	core	additional	discard	outcome
Be able to describe difference between area level indicators of socioeconomic status (SES) and individual level indicators of SES	5	2	3	HISG discuss
Health Inequalities aspects of common conditions such as	9	1	0	Core and

Obesity, Diabetes, Cardio-Vascular disease and mental health				amalgamated with one below
Graduates should be able be able to present a Global view of Health Inequalities, with case examples	6	3	1	AEW review wording and core
Graduates should be able to describe how different systems of health care impact on Health Inequalities	5	4	1	HISG discuss (amalgamated with one below)
Understanding of health policy and health systems in a wider context of society	7	3	0	AEW review wording and core
Graduates should understand the key principles of Primary Health Care and its essential role in reducing Health Inequalities	8	1	1	core
Benefits of continuity of care	4	3	2	Move to additional
the needs of economically deprived older people and young people in care	*5*	*2*	*2*	HISG discuss
Define 'cultural diversity' and apply this definition with respect to clinical practice	*8*	*1*	*1*	core
Critically appraise the use of key terms, such as race, ethnicity, culture, multiculturalism and inequalities of access to health care	*6*	*4*	*0*	core
List the different approaches there are to developing skills in meeting the needs of diverse populations and compare and contrast these	*3*	*6*	*0*	Move to additional
Describe existing equal opportunity legislation	*5*	*5*	*0*	HISG discuss

Explain how you would apply the legislation to your practice as a health care provider and as an employer	*2*	*7*	*1*	Move to additional
Evaluate the relevance of cultural diversity training in healthcare	*5*	*2*	*3*	Move to additional
An understanding of Equality and Human Rights legislation and how it overlaps with health inequalities	*6*	*4*	*0*	core

**Skills ILOs**	**Core**	**Additional**	**discard**	**outcome**
Evaluate institutional prejudices and how these relate to your own perspectives.	3	5	0	Move to additional
Identify strategies to challenge prejudice effectively and identify local policy in this area to ensure robustness.	4	6	1	Move to additional
Evaluate and justify the {above} approaches used in your own clinical practice.	4	3	2	Move to additional
Assess the impact (both positive and negative) of your attitudes on your clinical practice and demonstrate respect for patients and colleagues who encompass without prejudice, diversity of background and opportunity, language, culture and way of life.	7	2	1	core
Develop a generic approach to patients from diverse backgrounds, understanding that some patients require more input and advocacy than others.	9	0	1	core
Explain how you would apply Equalities legislation to your practice as a health care provider and as an employer	1	6	1	Move to additional

**Attribute ILO**	**core**	**additional**	**discard**	**outcome**
Empathy and compassion	10	0	0	AEW wording review and

				core
Evaluate your own attitudes and perceptions (including personal bias) of different groups within society.	10	0	0	core
trying to get students to challenge their own assumptions	6	1	1	HISG discuss
